# Invasion triple trouble: environmental fluctuations, fluctuation-adapted invaders and fluctuation-mal-adapted communities all govern invasion success

**DOI:** 10.1186/s12862-019-1348-9

**Published:** 2019-02-01

**Authors:** Kati Saarinen, Leena Lindström, Tarmo Ketola

**Affiliations:** Centre of Excellence in Biological Interactions, Department of Biological and Environmental Science, University of Jyväskylä, P.O. Box 35, FI-40014 Jyväskylä, Finland

## Abstract

**Background:**

It has been suggested that climate change will lead to increased environmental fluctuations, which will undoubtedly have evolutionary consequences for all biota. For instance, fluctuations can directly increase the risk of invasions of alien species into new areas, as these species have repeatedly been proposed to benefit from disturbances. At the same time increased environmental fluctuations may also select for better invaders. However, selection by fluctuations may also influence the resistance of communities to invasions, which has rarely been tested. We tested eco-evolutionary dynamics of invasion with bacterial clones, evolved either in constant or fluctuating temperatures, and conducted experimental invasions in both conditions.

**Results:**

We found clear evidence that ecological fluctuations, as well as adaptation to fluctuations by both the invader and community, all affected invasions, but played different roles at different stages of invasion. Ecological fluctuations clearly promoted invasions, especially into fluctuation mal-adapted communities. The evolutionary background of the invader played a smaller role.

**Conclusions:**

Our results indicate that climate change associated disturbances can directly increase the risk of invasions by altering ecological conditions during invasions, as well as via the evolution of both the invader and communities. Our experiment provides novel information on the complex consequences of climate change on invasions in general, and also charts risk factors associated with the spread of environmentally growing opportunistic pathogens.

**Electronic supplementary material:**

The online version of this article (10.1186/s12862-019-1348-9) contains supplementary material, which is available to authorized users.

## Background

Current climate change scenarios predict that in addition to the increase in temperature, fluctuations in temperature and other environmental conditions are also increasing [1] and creating selection pressures for biota. Some species benefit from these changes, and invasions and range expansions have been documented for many taxa [2–4]. In particular, fluctuations in environmental conditions might lead to the evolution of invasive genotypes [5] aiding species invasions. Thus, global climate change, with increased environmental fluctuations, could bring evolutionary and ecological problems to native fauna and flora: they must cope both with the direct changes caused by environmental fluctuations and with freshly-evolved invasive genotypes. This could be especially disastrous if native communities are mal-adapted to fluctuations.

One possible evolutionary explanation for the emergence of invasive species and genotypes is that they have evolved in a disturbed and fluctuating environment. It has been suggested that rapid fluctuations in particular, can select for traits that could promote invasion success, such as high population growth rate, plasticity and persistence [5–8]. Climate change has been suggested to lead to increased extreme events (e.g. [1]), but the pre-adaptive role of fluctuating environments on invasions has seldom been tested [5, 9]. The literature on invasions has been centered on the evolutionary background of the invader, but the community’s properties, such as diversity and relatedness with the invader can also influence invasion success [10]. The evolutionary background of the invader, the community’s pre-adaptations to fluctuations, as well as prevailing conditions in general, could also dictate the success of an invader. If a community is adapted to fluctuations, environmental fluctuations should not cause repercussions in population size and hence benefit invaders.

Although it can be argued that all environments are potentially prone to invasions, significant variation exists in the sensitivity of different environments to invasions. Empirical evidence supports the theory that heterogeneous (both in space and time) and disturbed environments are more prone to invasions than stable environments [11–15]. Disturbances might facilitate invasions by altering community composition, species competitive interactions, free resources, ecosystem processes, and propagule supply [10]. Recent research has also recognized that invasions are interactions between the invader and the environment invaded, and as such the invasive traits might only make sense in certain environments [16–18]. Since disturbed environments can promote the evolution of invasive traits [5], studying the evolutionary background and pre-adaptations of the invader together with the effects of the current environment can also generate important information on the causes of invasions, especially in the context of current climate change [17]. For example; will the increased fluctuations predispose communities to an increased risk of invasions, and will the fluctuation-adapted invaders invade such environments with even greater likelihood, as is suggested in the anthropogenically induced adaptation to invade – hypothesis (AIAI; [19])?

To test these key ideas about the ecological and evolutionary determinants of invasions [17], we used several species of microbes that had evolved for 2.5 months in two different kinds of environments; under constant temperature and under fluctuating temperature. These species and strains allowed us to make unique combinations of the invader (*Serratia marcescens*) and the community (*Pseudomonas chlororaphis, Enterobacter aerogenes* and *Leclercia adecarboxylata*) in which evolutionary adaptations either matched, or not, the environmental conditions during the invasions. With microbes it is straightforward to test general ecological and evolutionary theories, which are not amenable to testing with higher organisms (e.g. [20]). Consequently, microbial experiments have also become more popular in invasion biology [21–27]. However, the effects of environmental fluctuations, other than in resources, on invasion success [12, 23] have rarely been tested [28]. The invader species *S. marcescens* is an opportunistic pathogen capable of infecting various species such as plants, corals, nematodes, insects, fish and mammals [29–31]. Hence, our experiment provides also an important test on the determinants of spread of pathogenic bacteria and diseases facing climate change induced fluctuations.

We hypothesized that if fluctuation in temperature is generally a driver of the evolution of invader genotypes [5, 9], experimental evolution in fluctuating environment should lead to bacterial clones that are more invasive. If instead fluctuations during invasion (i.e. on an ecological scale) lead to an overall improved invasion success it gives support to the idea that disturbance by various means enhances invasion success (see above for details, [11–15]). Moreover, if fluctuations increase the invasion success of fluctuation-adapted clones in particular, that would lend support to the anthropogenically induced adaptation to invade – hypothesis (AIAI; [19]). This hypothesis postulates that human induced, often disturbed, habitats would allow for the invasion of those genotypes that have previously adapted to such conditions. Moreover, we expect that communities that have been evolving in fluctuating environments should be better in resisting invasions, and communities that had evolved in constant environments should be especially vulnerable to invasions. We explore the determinants of invasions over several time points to reveal if certain mechanism have especially predominant role in early stages of invasion (e.g. colonization/arrival stage), in comparison to latter stages of invasion (e.g. establishment).

## Methods

All the bacterial clones used in this experiment are from an evolution experiment [32], where we reared replicated bacterial populations (*n* = 10) of 9 different species separately (totalling 90 populations) at either a constant (30 °C) or thermally fluctuating regime (20–30 - 40 °C, at 2 h interval). The bacterial populations evolved in wells of a 100-well Bioscreen C® (Growth curves Ltd., Helsinki, Finland) spectrophotometer plate in thermal cabinets ILP-12, Jeio Tech, Seoul, Korea). The experiment lasted 79 days, and every three days 40 μl of culture was renewed to 0.4 ml of fresh Nutrient Broth medium (hereafter NB: 10 g of nutrient broth (Difco, Becton & Dickinson, Sparks, MD) and 1.25 g of yeast extract (Difco) in 1 l of sterile ddH20). After the experiment, we extracted 4 clones (i.e. colony forming units) from each bacterial population, utilizing dilution series plating, and stored the clones at − 80 °C. This experiment yielded a total of 80 clones from each of the species (totalling 720 clones). *Serratia marcescens ssp. marcescens* (ATCC® 13880™) was chosen as the invader because its ability to break DNA allowed easy recognition from other species using simple plating techniques [33, 34]. The 3 community species used in this study showed relatively high resistance against the invading *S. marcescens*, when reared together: *Pseudomonas chlororaphis* ATCC® 17418™, *Enterobacter aerogenes* ATCC® 13048™ and *Leclercia adecarboxylata* ATCC® 23216™ [34].

### Invasion experiment

**Fig. 1:**
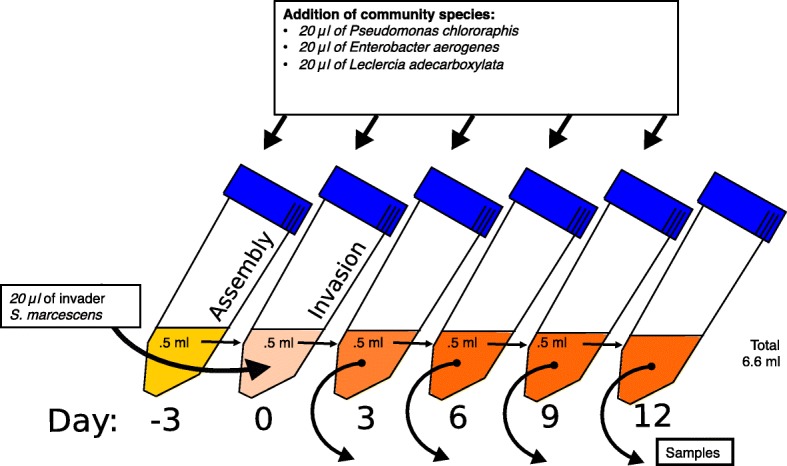
Overview of the setup of the invasion experiment. Invasions started with the three-day “assembly” period for the community. Invasion occurred three days later. Renewal to new tubes and sampling occurred every three days. This procedure was repeated with invaders adapted to fluctuating or constant temperature making invasion against community adapted to fluctuating or constant temperature in constant or fluctuating environment. Each of the eight combinations was replicated 10 times

As we were interested in the effects of invasion environment (constant vs. fluctuating temperature), evolutionary background of the invader (evolved in constant vs. fluctuating temperature) and evolutionary background of the community species (evolved in constant vs. fluctuating temperature) on the invasion success of *S. marcescens*, we set up an experiment testing all 3 effects together. We had 2 different thermal environments where the invasion took place: constant (30 °C) and rapidly fluctuating (2 h 20 °C - 2 h 30 °C - 2 h 40 °C) and community members had either evolved in constant environment or in fluctuating environment. Moreover, in these very same conditions we tested how clones of *S. marcescens,* that had evolved in independent replicate populations (*n* = 10), either in fluctuating or constant temperatures, were able to successfully invade. The experiment was initiated by allowing the community species an assembly / establishment period prior to invasion (Fig. 1). Community species were mixtures of clones from 10 independently evolved populations evolved either in constant or in fluctuating environments (previously mixed and frozen at − 80 °C (1:1 in 80% glycerol)). After thawing, 20 μl of each species clone mix (totalling 60 μl) was pipetted into experimental 15 ml centrifuge tubes (Sarstedt, Numbrecht, Germany) filled with 6 ml of NB, and tubes were placed in thermal treatments: at constant 30 °C or at fluctuating 2 h 20 °C - 2 h 30 °C - 2 h 40 °C (thermal cabinets: ILP-12, Jeio Tech, Seoul, Korea). Centrifuge tube caps were left loose to allow airflow. Throughout the experiment the cultures were stationary.

After the three-day community assembly period we renewed the resources by pipetting a 500 μl sample of each community into new tubes filled with 5.5 ml of NB. To maintain all community species in the community we supplemented cultures with 20 μl of each community species clone mix (the clones, before mixing, were also individually grown beforehand for 3 days at 30 °C, 60 μl of bacteria in 6 ml of NB in 15 ml centrifuge tubes). The added clone mixes had always the same evolutionary background as the community species (Fig. 1).

After renewal and species supply, invasions were initiated to create the 8 different environment - evolutionary background combinations. The 20 (10 from fluctuating and 10 from constant environment) *S. marcescens* invader clones had been propagated beforehand for 2 days at 30 °C (60 μl of bacteria in 6 ml of NB in 15 ml centrifuge tubes). To each community, we added 20 μl of *S. marcescens* invader clones, which corresponds to 4% of the renewed community (500 μl) and is equivalent with the amount of external gene flow from each community species (see above). After which the communities, now with an invader, were placed in thermal chambers. The rationale for species supplementation is the very effective invasion by *S. marcescens.* In un-supplemented system *S. marcescens* dominate the whole community within few days. Unfortunately, the supplementation renders detailed work on community’s responses to invasion useless.

The communities were propagated for 12 days after the invasion, and we renewed the communities (as above), added the gene flow from the community species (as above) and froze samples of each community to − 80 °C (final concentration of 40% glycerol) every 3 days (3, 6, 9 and 12 days after the invasion). After the experiment we plated all the community samples from all the time points (3, 6, 9 and 12 days after invasion, altogether 320 samples). We used a standard dilution series technique to achieve a 10^5^-fold dilution series that allowed the counting of separate colonies on agar plates. We plated the samples on DNase test agar with methyl green (Becton and Dickinson and Company, Sparks, MD; premade at Tammer-tutkan maljat, Tampere, Finland) that enabled the separation of *S. marcescens* from the community species (see: [33, 34]). The fate of specific community species was not monitored during the invasion experiment, due to frequent supplementation of species (see above).

### Data-analysis

To test if the experimental environment, evolutionary background of the invader, and evolutionary background of the community species had an effect on the invasion success of the invader (proportion of invaders), we used a generalized mixed model with a binomial error distribution and a logit-link implemented in stan glmer function in Rstan package in R. These kinds of analyses are highly preferred for percentage data instead of the linear models on arcsine square root transformed data [35]. As explanatory variables we fitted experimental environment, evolutionary background of the invader and evolutionary background of the community species (constant vs. fluctuating in all cases), and all possible interactions of the factors, with weakly informative priors. Since we measured the same invader clone against two different recipient communities (evolved in fluctuating or constant) and in two different invasion environments (fluctuating and constant), we fitted the identity of the invader clone as a random factor in the models to control for the non-independency of observations. Due to interactions between time and treatment levels we decided to split analysis based on time steps in order to facilitate interpretation of the results.

## Results

**Fig. 2:**
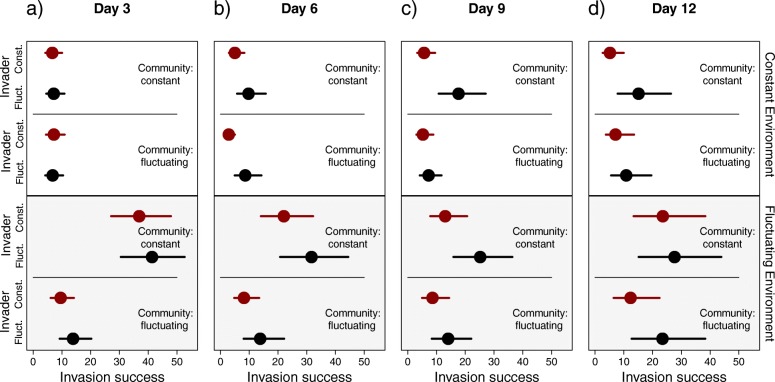
Invasion success due to evolutionary background and environment. Proportion of *S. marcescens* colonies during invasion in bacterial communities (**a**) three (**b**) six, (**c**) nine and (**d**) twelve days after the invasion, when invasion occurred either in constant or in fluctuating thermal conditions, or if invader or community evolved in fluctuating or constant conditions. Dots are medians of posterior distribution of estimates for treatment combinations. Whiskers denote 95% credible intervals. Pairwise tests for treatment level combinations can be found from the Additional file 1: Table S2

After three days of invasion, environment during invasion and evolutionary background of community, and their interaction explained strongly the invasion success (Table 1, Fig. 2). Regardless of the community’s evolutionary background the invasion was always strongest when the environment during invasion was fluctuating (in all pairwise comparisons *p* < 0.001, Table 2) compared to the constant environment. However, much of the significant main effect of the community was a result of a disproportionally large role of the fluctuating environment during invasion causing communities adapted to constant environments (i.e. maladapted to fluctuations) to suffer from invasions, compared to the fluctuation-adapted communities (*p* < 0.001, Table 2, Fig. 2 & Additional file 1: Figure S1.). In contrast, in the constant environment, the invasion success to fluctuation-adapted communities was comparable to the invasion success to communities adapted to constant environment (*p* = 0.972, Table 2).

**Table 1:** Effects of invader’s and community’s evolutionary background (fluctuating or constant thermal environment), and environment (fluctuating or constant) during invasion and their interactions on invasion success of Serratia marcescens, at third, sixth, ninth, and twelfth day of invasion

	Day 3				Day 6			
		95% CI				95% CI		
Effect	Estimate	lower	upper	*p*	Estimate	lower	upper	*p*
Intercept	32.904	26.295	40.806	<0.001	25.716	18.132	35.503	<0.001
Invader	2.172	-5.361	9.676	0.57	6.587	-0.914	15.385	0.086
Community	13.665	10.924	16.648	<0.001	8.705	6.315	11.511	<0.001
Environment	18.581	14.984	22.401	<0.001	12.396	8.979	16.215	<0.001
Invader by Community	0.213	-2.831	3.099	0.87	0.802	-1.846	3.193	0.544
Invader by Environment	2.149	-1.6464	5.999	0.278	1.221	-2.347	4.531	0.461
Community by Environment	13.717	10.985	16.724	<0.001	7.061	5.013	9.375	<0.001
3-way	0.251	-2.555	3.243	0.867	1.266	-0.882	3.426	0.243
	Day 9				Day 12			
		95% CI				95% CI		
Effect	Estimate	lower	upper	p	Estimate	lower	upper	p
Intercept	24.503	17.215	33.723	<0.001	31.72	20.908	46.328	<0.001
Invader	8.064	0.659	16.468	0.032	7.088	-5.822	20.381	0.234
Community	6.563	4.482	9.106	<0.001	4.409	2.53	6.586	<0.001
Environment	6.268	4.352	8.625	<0.001	12.201	8.326	16.64	<0.001
Invader by Community	4.192	2.091	6.584	<0.001	0.203	-1.853	2.245	0.846
Invader by Environment	0.938	-1.162	3.007	0.368	0.273	-4.27	4.23	0.888
Community by Environment	1.126	-0.217	2.509	0.094	3.213	1.14	5.582	0.003
3-way	0.805	-0.42	2.277	0.206	3.4	1.444	5.809	<0.001

Estimate indicates median of posteriori distribution of the estimate b. b=X^-1^y, where X is design matrix and y is posterior estimate at each level of treatment combinations. 95% credible intervals indicate variation around the estimate. p = propability of overlap with zero

**Table 2:** Post hoc comparisons of the treatment level combinations of environment during invasion and evolutionary background of community

Community’s evolution:			Constant	Fluctuating	Constant	Fluctuating
		Environment:	Constant	Constant	Fluctuating	Fluctuating
*3 days after invasion*						
	Constant	Constant	7.07			
	Fluctuating	Constant	0.924	7.15		
	Constant	Fluctuating	< 0.001	< 0.001	39.55	
	Fluctuating	Fluctuating	< 0.001	< 0.001	< 0.001	11.83
*6 days after invasion*						
	Constant	Constant	7.18			
	Fluctuating	Constant	0.002	5.14		
	Constant	Fluctuating	< 0.001	< 0.001	26.85	
	Fluctuating	Fluctuating	< 0.001	< 0.001	< 0.001	10.91
*9 days after invasion*						
	Constant	Constant	10.44			
	Fluctuating	Constant	< 0.001	6.35		
	Constant	Fluctuating	< 0.001	< 0.001	18.67	
	Fluctuating	Fluctuating	0.325	< 0.001	< 0.001	11.33
*12 days after invasion*						
	Constant	Constant	9.11			
	Fluctuating	Constant	0.782	8.92		
	Constant	Fluctuating	< 0.001	< 0.001	25.61	
	Fluctuating	Fluctuating	< 0.001	< 0.001	< 0.001	17.32

Treatment combinations of environment, and community evolution are indicated by the first two columns, and rows. In each submatrix, diagonal rows denotes median invasion success (%) in a given treatment, whereas off diagonal rows give propability of rank order change in posteriori estimates between treatment combinations

At six days after the invasion, fluctuating environment during invasion increased the invasion success (Table 1, Fig. 2). In addition, community’s evolution in constant environment led always to higher invasion success (Table 1) compared to communities evolution in fluctuating environment. However, the effect of community’s evolutionary background was more clear if invasion occurred at fluctuating environment (*p* < 0.001), than if it occurred in constant environment (*p* = 0.005) (Table 2).

At nine days after the invasion, environment remained the clearest denominator of invasion success (Table 1, Fig. 2), as in fluctuating environments the invasion was stronger (*p* < 0.001) than in constant environment. Also communities, that had evolved in constant environment, compared to communities evolved at fluctuating environment, suffered from the invasions the most (*p* < 0.011, Table 2). Invader evolution at fluctuating environment was found to increase invasion success compared to invader evolution at constant environment, against community that had evolved in constant environment (*p* = 0.007). However, effect of invader’s evolutionary background on invasion success was not detected (*p* = 0.207) if community had evolved in fluctuating environment (Additional file 1: Table S1).

Twelve days after invasion, the environment during invasion remained a good predictor for the invasion success (Table 1, Fig. 2). This was true in all comparisons despite significant three-way interaction (Table 1, Additional file 1, Table 2). Community’s evolution at constant environment predisposed it to stronger invasions (*p* < 0.001), except if environment was fluctuating during invasion and also if invader had evolved at fluctuating environments (*p* = 0.08) (Additional file 1: Table S2). Invader evolution increased invasion success but only when environment was constant and community had evolved in constant environment (*p* = 0.021, all other comparisons *p* > 0.12)(Additional file 1: Table S2).

## Discussion

As climate change increases disturbances [1] it is possible that invasions, range expansions, and evolution of invasive genotypes will become more common [4, 5, 9, 10, 19, 36]. We tested if evolution in a fluctuating environment, and fluctuations during invasions, affect the invasion probability of an environmentally growing opportunistic pathogen (*S. marcescens*) by using experimentally evolved strains of bacteria [32]. Based on our results it was clear that invasions were promoted under fluctuating environments and especially if community species had not adapted to the fluctuating environment. However, the role of invader evolution on invasions was less profound.

Throughout the experiment, environmental fluctuations played a major role in facilitating invasions. However, especially at the early stages (three days after invasion, Fig. 2a) of invasion, in the constant environment the invasion success was low, regardless of the invader’s or community’s evolutionary background. Thus, it seems that fluctuations reveal, and constant environments buffer, evolutionary differences of strains. In the framework of current climate change this finding suggests that invasions will be increasing due to increased fluctuations. Our paper is the first to report that thermal fluctuations are able to cause similar increases in invasions as resource level fluctuations manipulated in other studies [11–15].

However, it was also evident that increasing fluctuations do not treat all communities equally. Throughout the experiment, the communities that were mal-adapted to fluctuating environments experienced the strongest invasions (Fig. 2a, Table 2). This is an interesting and novel finding as most of the research has emphasized the role of invader background on invasion success [5, 9, 10] rather than the community’s properties. This finding is akin to the anthropogenically induced adaptation to invade – hypothesis (AIAI; [19]), which suggests that human altered habitats, often considered disturbed, would be more vulnerable to invading species. However, instead of invaders being pre-adapted to such conditions, it seems that fluctuations or disturbances can also operate via mal-adapted communities. Thus, if climate change brings increased fluctuations to areas with a previous history of fairly constant conditions, these areas should be more vulnerable to invasions.

We did not find that evolution in fluctuating temperature pre-adapted the invader strains to be especially good at colonizing (three days after invasion) when environments fluctuated [37, 38]. Although, environmental fluctuation is one of the candidates for the evolution of invasive genotypes [5, 9, 10], invader’s evolutionary background had, at best, marginal effects, however pointing towards increased invasiveness after evolving in fluctuating environments (Day 6, Table 1). Weaker effects of the invader’s properties could also partially stem from our experimental setup. For example, originally rather small [32] evolutionary differences found in few traits that are potentially important to invasion, could lead to small invader effects. However, in our data it was also evident that at one time point (9 days after the invasion) the invaders evolved in fluctuating environments were found to be good at invading communities adapted to constant environments. Such difference was absent if community had evolved at fluctuating environment. Thus, small role of invader evolution on invasion might not be explained by overall small evolutionary effects, but by complex interactions between other factors affecting invasions. Due to supplementation of community members, to allow existence of species with strong invader, we did not follow individual species, as their population sizes were strongly affected by supplementation. It could well be that single community species, or smaller group of species, is responsible for the resistance of invasion, instead of community as a whole. However, with our system, this is hard to resolve.

Strong interactions between the community’s evolutionary background, invasion environment and at latter stages also invader’s evolutionary background, confirms that a large part of invasions can be understood by taking into account complex interactions between ecological conditions and evolutionary adaptations [16–18]. Therefore, it is important to bear in mind that the environment and genotypes always play a role and invasion success is not solely dependent on the properties of an invader [5, 9, 10]. This could mean that the quest for identifying potential invaders could be a rather weak way to mitigate invasion threat, as invasions can be dictated by environmental conditions in interplay with a community’s adaptations to environmental conditions. However, based on our results, mapping areas with largest risks of mal-adapted populations, perhaps small and isolated populations experiencing large and rapid environmental changes could be a fruitful avenue for prediction and mitigation of invasion risks.

## Conclusions

To summarize, we found a large role of environmental fluctuations in aiding invasions throughout our 12 day long experimental invasion. Moreover, during the first days, environmental fluctuations also revealed a novel effect of the evolutionary background of the community on invasion success, as communities that evolved in a constant environment were especially vulnerable to invasion in the fluctuating environment. This means that climate change may cast a two-fold disadvantage for those communities that are poorly adapted to environmental fluctuations. Throughout the experiment, environment clearly remained the biggest cause of invasions. A community’s evolution in a constant environment increased the invasion risk at the early stages, and the role of the community gradually decreased during the experiment. Invader evolutionary played also a role but in complex interactions with invader and environment. From our experiment it is clear that thermal fluctuations threaten native populations and communities in many ways – via ecological phenomena, by facilitating invasions in general, and in evolutionary ways via fluctuation-adapted invaders and fluctuation mal-adapted communities. Our novel experiment, manipulating all key players involved in invasions in climate change altered conditions, offers not only a test for the general evolutionary theories, but also reveals important insights on determinants of bacterial pathogen prevalence and invasion under climate change induced conditions.

## Additional file

Additional file 1: For: Saarinen, Lindström, Ketola (EVOB-D-17-00372). Invasion triple trouble: Environmental fluctuations, fluctuation-adapted invaders and fluctuation-mal-adapted communities all govern invasion success. **Figure S1.** Invasion success of *S. marcescens* in each replicate population during time, grouped by environment and invader evolutionary history. Line colours indicate if community had evolved at constant environment (blue) or at fluctuating environment (red). **Table S1.** Post hoc comparisons of treatment level combinations for invader evolutionary background by community evolutionary background interaction at nine days after invasion. **Table S2.** Post hoc comparisons of all treatment level combinations from model containing timepoints three, six, nine and twelve days after invasion. Treatment combinations of environment, invader evolution and community evolution are indicated by the first three columns, and rows. Each submatrix denotes dataset used to derieve estimates. In each submatrix, diagonal invasion success in given treatment, whereas off diagonal give propability of rank order change in estimates between treatment combinations. (DOCX 119 kb)
