# Supplementary material for: Invasion triple trouble: environmental fluctuations, fluctuation-adapted invaders and fluctuation-mal-adapted communities all govern invasion success
Source: BMC Evol Biol. 2019 Feb 1;19:42. doi: 10.1186/s12862-019-1348-9 (PMC6359858; doi:10.1186/s12862-019-1348-9)
Supplement: Supplementary file 1 — For: Saarinen, Lindström, Ketola (EVOB-D-17-00372). Invasion triple trouble: Environmental fluctuations, fluctuation-adapted invaders and fluctuation-mal-adapted communities all govern invasion success. Figure S1. Invasion success of S. marcescens in each replicate population during time, grouped by environment and invader evolutionary history. Line colours indicate if community had evolved at constant environment (blue) or at fluctuating environment (red). Table S1. Post hoc comparisons of treatment level combinations for invader evolutionary background by community evolutionary background interaction at nine days after invasion. Table S2. Post hoc comparisons of all treatment level combinations from model containing timepoints three, six, nine and twelve days after invasion. Treatment combinations of environment, invader evolution and community evolution are indicated by the first three columns, and rows. Each submatrix denotes dataset used to derieve estimates. In each submatrix, diagonal invasion success in given treatment, whereas off diagonal give propability of rank order change in estimates between treatment combinations. (DOCX 119 kb) [file 12862_2019_1348_MOESM1_ESM.docx]

**Additional file 1.** For: Saarinen, Lindström, Ketola (EVOB-D-17-00372). Invasion triple trouble: Environmental fluctuations, fluctuation-adapted invaders and fluctuation-mal-adapted communities all govern invasion success"

**Figure S1.** Invasion success of *S. marcescens* in each replicate population during time, grouped by environment and invader evolutionary history. Line colours indicate if community had evolved at constant environment (blue) or at fluctuating environment (red).

**Table S1.** Post hoc comparisons of treatment level combinations for invader evolutionary background by community evolutionary background interaction at nine days after invasion.

**Table S2.** Post hoc comparisons of all treatment level combinations from model containing timepoints three, six, nine and twelve days after invasion. Treatment combinations of environment, invader evolution and community evolution are indicated by the first three columns, and rows. Each submatrix denotes dataset used to derieve estimates. In each submatrix, diagonal invasion success in given treatment, whereas off diagonal give propability of rank order change in estimates between treatment combinations.
